# Preferences and Perceptions of Training Models in Global Surgery With a Focus on Orthopedics: A Scoping Review

**DOI:** 10.1007/s12178-026-10016-z

**Published:** 2026-03-11

**Authors:** Harlene Kaur, Daniel Flynn, Andrew Haggarty, Sanjeev Sabharwal

**Affiliations:** 1 University of Massachusetts Chan Medical School, 55 N Lake Ave, Worcester, MA 01655 USA; 2 UCSF Benioff Children’s Hospital Oakland - Pediatric Orthopaedics, 744 52Nd St, Oakland, CA 94609 USA; 3 Institute of Global Orthopaedics and Traumatology (IGOT), Department of Orthopaedics, University of California, San Francisco, USA

**Keywords:** Global orthopaedic education, Low- and middle-income countries, Surgical training exchanges, Learner perceptions, Virtual and hybrid education, Capacity building

## Abstract

**Purpose of Review:**

This scoping review examines learner-reported preferences and experiences with remote learning, observership, and visiting-surgeon exchange models in low-resource settings. We outline potential benefits, limitations, equity considerations, and evidence gaps that can inform ethical and sustainable global orthopaedic education partnerships.

**Recent Findings:**

Recent literature on global orthopaedic education has largely focused on training exchanges involving high-income country (HIC) trainees in low- and middle-income countries (LMICs), with reported benefits including skills transfer and professional development alongside challenges related to continuity of care, resource burden, and limited reciprocity. However, despite growing consensus around equity and reciprocity, the perspectives of orthopaedic trainees and practicing surgeons in low-resource settings remain underrepresented in the current literature.

**Summary:**

Learners in low-resource settings valued international exchanges that provided structured teaching, subspecialty exposure, and access to higher-volume clinical environments. Virtual and simulation-based education demonstrated consistent improvements in knowledge and surgical skills despite technical and cost-related barriers. In-person and hybrid programs were associated with meaningful capacity-building benefits but required substantial resources, infrastructure, and long-term institutional commitment. Across all models, challenges related to infrastructure, cost, equity, and reciprocity remained. Notably, none of the included studies examined LMIC learner perspectives on bidirectional exchange with higher-resource settings, despite growing emphasis on reciprocity in contemporary partnership frameworks. These findings underscore the need for locally driven, sustainable global orthopaedic education partnerships with stronger outcome evaluation and greater LMIC leadership.

## Introduction

Global interest in international orthopaedic training exchanges has expanded rapidly over the past two decades, particularly among trainees from high-income countries (HICs) seeking clinical and educational experience in low- and middle-income countries (LMICs) [1, 2]. As defined by the World Bank, country income classifications are based on gross national income per capita, with HICs corresponding to high-income economies and LMICs encompassing low-, lower-middle, and upper-middle income economies [3]. These programs are often motivated by a desire to enhance surgical capacity, address disparities in musculoskeletal care, and promote global collaboration [1]. However, perspectives from host institutions in LMICs reveal a more nuanced reality. LMIC surgeons frequently report that visiting trainees contribute more meaningfully to education and knowledge exchange than to long-term capacity building, and that short-term rotations can sometimes impose administrative or resource burdens on already strained health delivery systems [1, 2]. Reported challenges include limited reciprocity, unequal access to learning opportunities, racial and cultural tension, and competition for operative cases amongst trainees from HICs and LMICs [2, 4].

Across the broader surgical literature, similar themes emerge [2, 5]. Systematic reviews synthesizing LMIC host institutions and individuals’ perspectives on visiting trainees and physicians from HICs describe both benefits, such as relief of clinical workload, skills transfer, and career advancement, and drawbacks, including poor continuity of care, ethical concerns, and insufficient follow-up [5]. Collectively, these findings underscore a persistent imbalance between HIC-driven objectives and LMIC-defined priorities, highlighting the need for partnership models that are equitable, bidirectional, and sustainable.

Recent frameworks have attempted to operationalize these principles. The Academic Model Providing Access to Healthcare (AMPATH) has demonstrated that long-term, bidirectional exchange grounded in shared governance and mutual benefit can foster enduring educational capacity [6]. For instance, the Institute for Global Orthopaedics and Traumatology (IGOT) model at University of California San Francisco (UCSF) emphasizes sustainable collaboration by building academic partnerships, remote education and bidirectional learning opportunities, and joint research endeavors to strengthen local training environments in LMICs [7]. Building on these examples, a seven-domain framework for global orthopaedic outreach delineates professional development, governance, finance, and community impact as measurable pillars of partnership sustainability [8]. Contemporary consensus statements further advocate for formalized academic agreements, local leadership, and reciprocal innovation to dismantle hierarchical dynamics and highlight LMIC expertise [9, 10].

Despite these advances, most existing research focuses on visiting HIC trainees and host institutions, with comparatively little attention to learners within low-resource settings. It remains unclear how orthopaedic trainees and practicing surgeons in LMICs perceive different training models, such as remote educational initiatives, short-term clinical observerships, and visiting-surgeon programs, as well as how they value horizontal collaborations, defined as peer-level exchanges between institutions with comparable resource settings, versus vertical collaborations, which involve partnerships with higher-resource institutions or more senior external faculty. Understanding these perspectives is essential to inform future partnerships that are both context-appropriate and learner-driven.

Accordingly, this scoping review aims to evaluate how orthopaedic and other surgical learners in low-resource settings describe their preferences and experiences with different educational models when interacting with HIC programs. Specifically, we planned to explore perceived benefits, limitations, and equity considerations across remote learning, clinical observership, and visiting-surgeon exchange models, as well as any differences in reported priorities between postgraduate trainees and practicing surgeons from LMICs. By mapping current evidence and identifying knowledge gaps, this review seeks to further guide the design of ethical, sustainable, and learner-centered global orthopaedic education partnerships.

## Methods

This scoping review was conducted to characterize preferences and perceptions of international training models among orthopaedic and surgical learners in low-resource settings. The review followed the Preferred Reporting Items for Systematic Reviews and Meta-Analyses extension for Scoping Reviews (PRISMA-ScR) guidelines [11]. A medical librarian (A.H.) assisted in developing and refining the search strategy.

### Search Strategy

Comprehensive searches were performed in Medical Literature Analysis and Retrieval System Online (MEDLINE) via PubMed and Ovid, Cumulative Index to Nursing and Allied Health Literature (CINAHL), and Education Resources Information Center (ERIC) via EBSCO, Scopus (Elsevier), and the Cochrane Central Register of Clinical Trials from database inception through September 2025. Search terms reflected four core constructs: (1) medical or clinical education, (2) remote or in-person instruction/observerships/exchange programs, (3) LMICs or low-resource settings, and (4) learner perceptions or experiences. Full database specific search strings are provided in Supplementary Appendix A.

### Study Selection

All identified records were imported into Covidence [12] (Veritas Health Innovation, Melbourne, Australia) for duplicate removal and screening. Title and abstract screening, followed by full-text review, was performed independently by two reviewers (H.K. and D.F.), with disagreements resolved by a third reviewer (S.S.). Studies were eligible for inclusion if they met all of the following criteria: (1) involved orthopaedic or surgical learners based in an LMIC or low-resource environment; (2) reported on experiences, perceptions, or preferences regarding an educational exchange or training model (remote learning, in-person observership, visiting-surgeon program, horizontal, vertical, or hybrid training, defined as programs integrating both virtual educational components and in-person clinical or surgical experiences); and (3) were available in English. Studies involving mixed surgical specialties were included when orthopaedic learners were part of the study population or when findings addressed general surgical education models applicable to musculoskeletal care. Editorials and commentaries were included if they directly reported LMIC learner perspectives or preferences. Conference abstracts without full text and studies focused exclusively on high-income country trainees were excluded.

### Data Extraction and Synthesis

Data extraction was performed in Covidence by two reviewers (H.K. and D.F.). Extracted variables included the country sponsoring the education and training program and the country where the education and training was conducted, surgical specialty, learner population, sample size, training level, structure of the educational model, format of instruction (virtual, in-person, hybrid, horizontal, or vertical), and reported learner perceptions. When available, funding sources and conflict-of-interest disclosures reported in each article were extracted to characterize the financial structures underlying these initiatives. Findings were synthesized qualitatively because of heterogeneity in study designs, outcome measures, and reporting of educational effects.

## Results

### Search Results

**Fig. 1:**
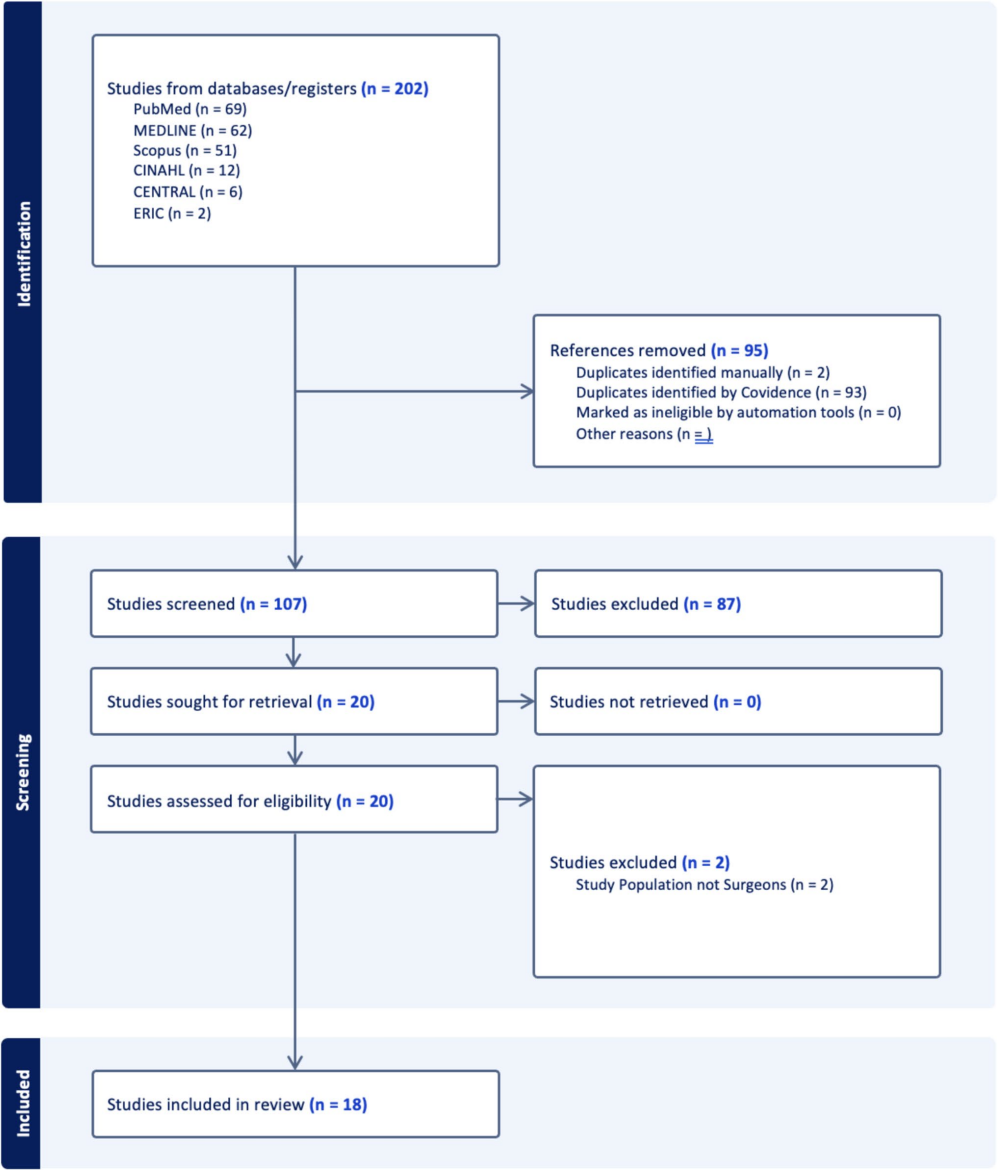
PRISMA (Preferred Reporting Items for Systematic Reviews and Meta-Analyses) flow diagram. Flow diagram illustrating the identification, screening, eligibility assessment, and inclusion of studies in this scoping review in accordance with PRISMA-ScR guidelines

**Fig. 2:**
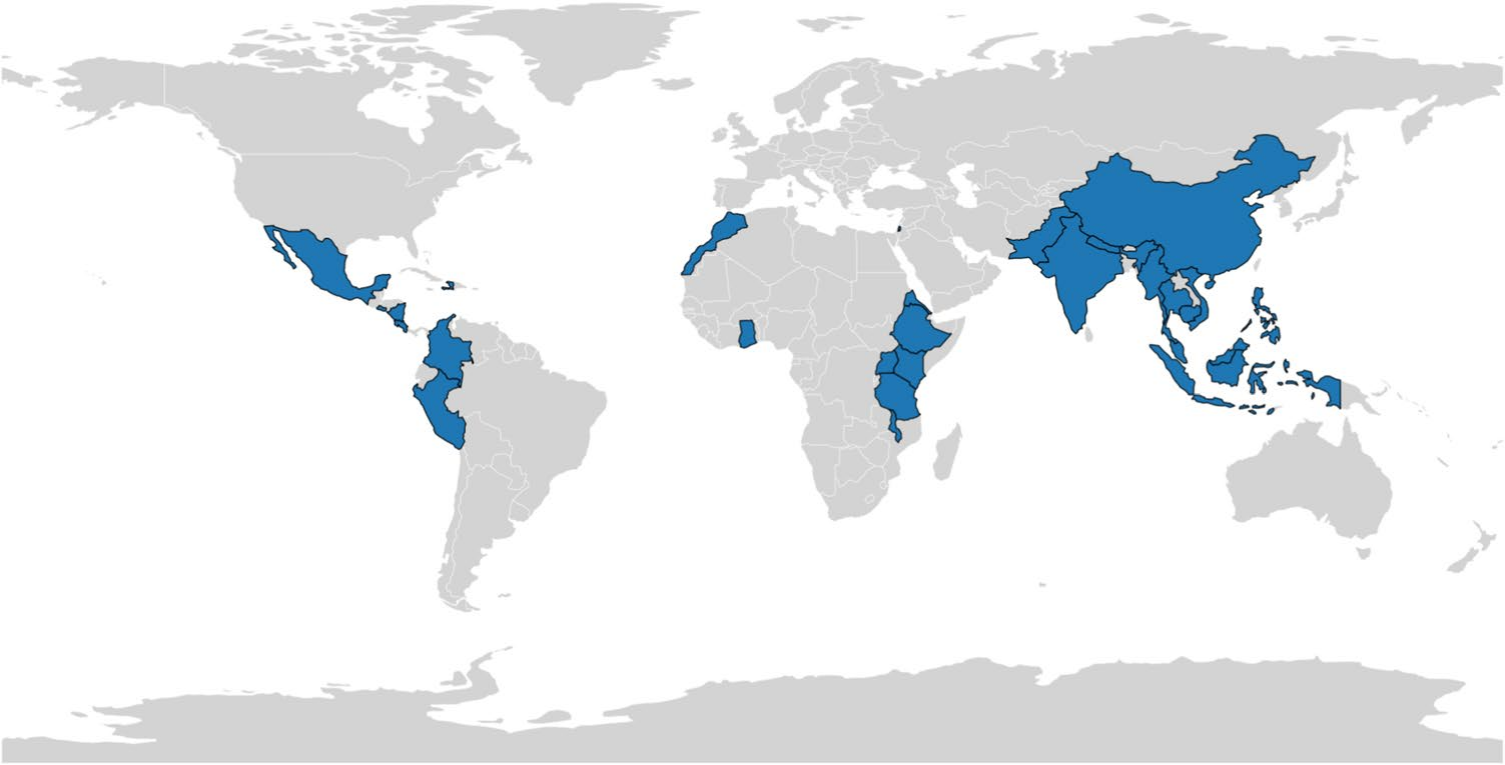
Geographic distribution of learner populations in included studies. Map illustrating the countries represented among orthopaedic and surgical learners in the 18 included studies, spanning low- and middle-income countries across multiple global regions

The search yielded 202 records. After removal of 95 duplicates, 107 studies underwent title and abstract screening. Twenty full-text articles were assessed, and 18 met inclusion criteria (15 data-based studies and 3 commentary papers; Fig. 1, Table 1). The included studies represented 14 LMICs (Fig. 2) and a range of surgical specialties, including orthopaedics, general surgery, neurosurgery, radiology, and otolaryngology.

**Table 1:** Summary table of 18 studies included

Author (Year)	Study design	Host Institution Location	LMIC Institution Location(s)	Learner Population	LMIC Learner Sample Size	Training Level	Study Aim
Agrawal 2014* [16]	Cross sectional study	India	India	Endocrine Surgeons	36	Attendings and Residents	Telemedicine for endocrine surgeon education
Asfaw 2023 [21]	Quasi-Experimental Study	USA	Costa Rica, Mexico	General Surgery	36	Attending and Resident	Virtual telesimulation laparoscopic training feasibility
Woodard 2024 [22]	Case series	USA	Indonesia	Radiology	13	Attendings Residents	VR ultrasound-guided biopsy training utility
Lepard 2020 [15]	Cross sectional study	USA	Southeast Asia LMICs (Myanmar, Indonesia, Cambodia, Thailand, and Nepal)	Neurosurgery	45	Residents	Neurosurgical training structure in Myanmar
Merrell 2007 [26]	Cohort study	USA	Vietnam	Microsurgery	> 200	Attending	Operation Smile microsurgery program review
Schonfeld 2013 [24]	Cohort study	Germany	Kenya	Ophthalmology	685	Residents	Intermittent visits establishing vitreoretinal surgery
Taekman 2017 [23]	Quasi-Experimental Study	USA	Uganda	Obstetrics, Anesthesia, Nursing	48	Attendings, Residents, Nurses	Simulation to improve Post-Partum Hemorrhage management confidence
Theodorakopoulou 2019 [20]	Cohort study	UK	Gaza	Orthopaedics, General Surgery, nursing	11	Residents	Tele-education in burn care
Jungehulsing 2019 [25]	Experience Report	Germany	Eritrea	Otolaryngology	Not Stated	Attendings, Residents, Nurses	International training experience in Medcare
Roberts 2021 [1]	Cross sectional study	USA	Multiple LMIC (Haiti, Nicaragua, Colombia, Ghana, Ethiopia, Kenya, Tanzania, Malawi, Nepal, Myanmar)	Orthopaedics	51	HIC Residents, LMIC Attendings and Residents	Resident motivations for LMIC rotations
Khan 2025* [13]	Cross sectional study	Pakistan	Pakistan	General Surgery, Bariatric Surgery, Gastrointestinal Surgery	55	Attendings and Residents	Barriers to bariatric surgery training
LaGrone 2017 [14]	Qualitative research	USA	Peru	Trauma Surgery and Emergency Medicine	50	Attendings	Impact of globalization on Peruvian training
Martins 2021* [17]	Quasi-Experimental Study	Pakistan	Pakistan	General Surgery	N/A	Residents	Peer-taught online research workshops evaluation
Moreira 2021 [27]	Cross Sectional Study	USA	Multiple LMIC (Mexico, El Salvador, Peru, Morocco, Pakistan, China, Malaysia, and the Philippines)	Neurosurgery, Radiation Oncology, Oncology, Pathology, Radiology	Online Course N = 36, In-Person Course N = 20	Attending	Hybrid pediatric neuro-oncology course design
Siddharthan 2021 [28]	Cohort study	USA	Uganda	Interventional Pulmonology	3	Attendings	Tele-bronchoscopy training pilot in Uganda
Quraishi 2021 [18]	Text and opinion	UK	Editorial	Otolaryngology	N/A	N/A	Challenges in LMIC otolaryngology training
Fagan 2021 [19]	Text and opinion	South Africa	Editorial	Otolaryngology	N/A	N/A	COVID-19 advancing digital surgical education
Costa 2021 [29]	Text and opinion	Italy	Editorial	Neurosurgery	N/A	N/A	Webinar and videoconference roles in COVID-19

* These studies represent within-country, multi-institutional educational models within a single LMIC, without involvement of an external country

Abbreviations: COVID-19: Coronavirus disease 2019; HIC: High-income country; LMIC: Low- and middle-income country; UK: United Kingdom; USA: United States of America; VR: Virtual reality; N/A: Not available.

### Learner Stated Preferences from LMIC

Four studies surveyed LMIC communities to characterize training needs and the perceived role of HIC partnerships [1, 13–15]. One study from Pakistan surveyed local surgery residents and consultants and identified key barriers to bariatric surgery development, including limited awareness among the public and surgeons, financial constraints, faculty shortages, societal stigma, and policy gaps [13]. Respondents identified vertical education through international exchange programs as the most favorably viewed strategy for strengthening surgical training, with international collaborations also receiving strong support. A report from Peru reported mixed perceptions of surgical globalization in the context of trauma care. Peruvian surgeons reported an eroded sense of agency and inadequate preparation to adapt international standards as negative consequences, while also recognizing positive effects such as improved access to research funding, advanced training opportunities, and quality improvement education that ultimately raised expectations for patient care [14]. Roberts et al. highlighted the discourse between HIC resident rotators and LMIC host surgeons in orthopaedic rotations. They stated that North American residents were primarily motivated by exposure to different case mixes, cultural immersion, and interest in global orthopaedics, whereas LMIC hosts valued the reciprocal exchange of knowledge, participation in high-volume centers, and the positive institutional impact of visiting residents [1]. A survey of Southeast Asian LMIC neurosurgeons identified limited case volume and research opportunities as key concerns within their home country. The authors emphasize that these gaps are a cause for seeking HIC partnerships, which should prioritize targeted exchange to address these deficiencies [15].

### Horizontal Models

A peer-led tele-education program linking endocrine surgeons from a tertiary academic center in India with peer surgeons and trainees at remote partner institutions across India demonstrated positive outcomes, with 94.4% of participants agreeing that tele-education enhanced knowledge exchange, 94.4% rating the experience as good to excellent, and 100% expressing interest in continuing tele-education in the future [16]. A proposed horizontal, peer-taught research education protocol for surgical residents in Pakistan anticipated that over 80% of participants would rate peer-led instruction as effective based on post-intervention feedback measures [17]. However, outcome data from this model were not reported. We were unable to identify any studies that investigated exchanges between two different LMICs.

### Vertical Models

#### Virtual Methods of Instruction

The benefits of virtual-based education were discussed in Qurashi et al. and Fagan et al. review and opinion papers, respectively [18, 19]. These authors reflected on how the coronavirus disease 2019 (COVID-19) epidemic has increased the level of web-based education in LMIC, benefiting learners in LMIC and giving a more equal opportunity to those available to HIC learners.

Four studies evaluated vertically structured virtual or simulation-based exchange models for surgical education in LMICs. One study implemented telemedicine educational conferences [20], while three separate groups examined virtual simulation-based learning models [21–23]. In Gaza, telemedicine education increased familiarity with evidence-based burn medicine principles and allowed surgeons to critically appraise information and apply it in their clinical practice. The study reported the success of this course by highlighting that after the course, the surgeons from Gaza performed equally as well on end-of-unit exams as those from HIC who took the class in person [20].

Simulation based studies further reinforced the value of virtual training. A simulation-based training curriculum demonstrated significant improvements in laparoscopic skills among each of the five domains tested (Peg transfer, circle cut, endoloop, extracorporeal knot, and intracorporeal knot) in Costa Rica and Mexico. Furthermore, 100% of participants found virtual simulation an effective learning tool and 98% wanted to continue learning [21]. Woodard et al. established the feasibility of using virtual reality simulation in ultra-sound guided breast biopsy in Indonesia, emphasizing the potential for safe, reproducible, standardized procedural instruction [22]. Likewise, a screen-based simulation reported an overall improved confidence in managing postpartum hemorrhage, with 100% of learners endorsing the training as effective [23]. However, these papers also reported certain challenges in virtual learning (Table 2). Several studies identified persistent barriers, including technical limitations with web-based conferences and simulation equipment [20–22], restricted practice time [20, 21], uncertainty regarding clinical transferability of simulation-based learning [22, 23], and high implementation costs associated with advanced virtual reality platforms [22].

**Table 2:** Potential opportunities and barriers in surgical education and training models between low- and middle income- and high-income countries

Learning Model	Opportunities	Barriers/Limitations
Horizontal (LMIC–LMIC)	High favorability among participants, Peer-led instruction enhanced knowledge exchange; high perceived effectiveness of peer-taught tele-education research curriculum	Lack of published reports between two LMIC institutions; Lack of implementation data
Vertical – Virtual	Increased access to education and equity in learning opportunities following COVID-19 expansion of web-based education; telemedicine in LMIC improved evidence-based knowledge with exam performance comparable to in-person HIC learners; simulation-based curricula demonstrated significant improvements in laparoscopic and procedural skills, with high learner endorsement	Technical limitations of web-based platforms; restricted practice time; uncertainty regarding clinical transferability of simulation-based learning; high costs of advanced VR platforms
Vertical – In-Person	Increased trainee operative participation and reduced preparation time; progressive transition to supervised independent practice; improved academic engagement of local surgeons; effective direct skill transfer	Resource-intensive and dependent on sustained HIC commitment; limited local infrastructure and equipment; challenges establishing subspecialty programs in resource-constrained settings
Hybrid (Virtual + In-Person)	Needs-based curriculum design with measurable improvement across multiple educational domains; reported improvements in clinical knowledge and changes in patient management following in-person procedural reinforcement	High cost and logistical complexity; resource intensiveness of procedural supervision; limited reporting of LMIC participant perceptions

Abbreviations: COVID-19: Coronavirus disease 2019; HIC: High-income country; LMIC: Low- and middle-income country; VR: Virtual reality.

#### In-Person Visitation

In contrast, three studies emphasized the role of vertical in-person education [24–26]. In a Kenya-Germany vitreoretinal surgery partnership between 2000–2010, trainee-performed surgeries in a Kenyan hospital rose from 29 to 73% and preparation time decreased by 60% [24]. Similarly, the Medcare program, which built a new hospital in Eritrea in 2005 and an otolaryngology clinic in 2010, allowed for in-person exchange. Following the establishment of these facilities, HIC otolaryngologists and anesthesiologists completed 25 missions to Eritrea, progressing from procedural demonstrations to supervision of local surgeons. The authors noted similar case pathology between Eritrea and Germany, which facilitated direct skill transfer, though outcomes and participant perspectives were not formally assessed [25]. Similarly, Operation Smile conducted an in-person surgical training program in microsurgery in Vietnam, consisting of an 8-day education, including 2 days of patient screening, 1 day for educational conferences, and 5 days for surgery. The program’s impact was reflected in the increasing academic engagement of local physicians, with Vietnamese surgeons presenting nearly half of the papers at mission-associated educational conferences held in Vietnam following the intervention [26].

Despite these demonstrated benefits, in-person HIC–LMIC training model also face substantial challenges. These partnerships require sustained long-term commitment and are often hindered by limited local infrastructure and inadequate equipment, though provision of supplies by HIC partners helped mitigate some of these barriers [24]. Furthermore, limited healthcare resources in LMICs impeded the establishment of resource-intensive subspecialty programs like microsurgery [26].

#### Hybrid: Virtual and In-Person

Two studies have evaluated a combination of virtual and in-person education for HIC-led surgical education in LMICs [27, 28]. The St. Jude Global Academy Neuro-Oncology Training Seminar used a needs assessment in 16 countries and then used those results to create a 9-week online course followed by a 7-day workshop in the United States. The course evaluated 5 different domains in neuro-oncology (barriers to care, possible solutions, the molecular classification of pediatric central nervous system tumors and its clinical implications). Results from pre-course and post-course tests evaluating course content showed that course participant performance improved in every educational domain tested. Furthermore, 65% of participants found they improved their knowledge in managing pediatric central nervous system (CNS) tumors and 90% reported that at least one aspect of their clinical care improved [27]. A hybrid instructional program for bronchoscopy initially utilized an online instructional program, and then HIC exchange in-person training in Uganda. The benefits of this training resulted in the 3 proceduralists in Uganda to change management in 9/14 cases experienced over the week due to the access of bronchoscopy. The perceptions of the LMIC proceduralists were not reported. High cost and resource intensiveness, as procedural teaching requires direct oversight were the major reported challenges of such a program [28].

#### Funding and Disclosures Reported in Included Studies

Reporting of funding sources varied substantially across the included studies, and many articles did not explicitly describe financial support for educational program development or evaluation. Three studies reported identifiable external funding mechanisms, including philanthropic, institutional, or government-affiliated support [22, 23, 28]. For example, Taekman et al. reported support from the Social Entrepreneurship Accelerator at Duke and the Duke Endowment, with the accelerator funded through a cooperative agreement with the United States Agency for International Development (USAID) [23], while Siddharthan et al. reported external grant support for bronchoscopy training from the Chest Foundation and the Association of Interventional Pulmonary Program Directors [28]. One study explicitly reported that no dedicated research funding supported the described educational activities [18], while 14 of the studies did not report funding information. In addition, two technology-focused studies reported industry or institutional conflict-of-interest disclosures related to program development, including an investigator-initiated study sponsored by an imaging industry partner [22], and a simulation platform with declared proprietary interest [23].

## Discussion

This scoping review explored how surgical learners in low-resource settings describe their experiences with and preferences for different educational and training models. Across 18 studies spanning a diverse group of LMICs, surgical specialties, and program structures, several themes emerged. Learners frequently emphasized the value of structured teaching, subspecialty expertise, and exposure to higher-volume clinical environments, which were most often associated with vertical collaborations [20–23, 26, 30]. Horizontal partnerships, while less commonly described, were viewed positively when tailored to local needs [16, 17]. Virtual and simulation-based education expanded access to training but was constrained by infrastructure limitations, cost, and uncertainty regarding clinical translation [20–22]. In-person and hybrid programs supported hands-on skill development and increased surgical autonomy but required sustained engagement and substantial resources [25–28, 30]. Across models, concerns related to equity, local autonomy, and reciprocity were recurrent [14, 15]. Together, these findings highlight key considerations for the design and evaluation of global surgical education partnerships.

### Structured Training Through Vertical Collaborations

Across studies, learners in LMICs consistently emphasized the importance of access to structured teaching, subspecialty expertise, and higher-volume clinical exposure. Vertical collaborations, especially those involving direct instruction from higher-resourced HIC centers, were often described as valuable for technical skill development and exposure to evidence-based surgical practice [20–23, 26, 30]. These preferences reflect the ongoing mismatch between the large burden of musculoskeletal disease and the limited surgical training opportunities available in many low-resource environments. Based on our review, there was little difference between the perspectives of postgraduate trainees and practicing surgeons in LMICs, suggesting that these educational priorities remain constant across different stages of the surgical career [1, 13–15].

### Horizontal Collaborations and Regional Capacity-Building

Horizontal collaborations were less frequently reported but were well received where implemented. Examples such as peer-led tele-education among endocrine surgeons from India and research instruction for residents in Pakistan were viewed as practical and relevant to local educational needs [16, 17]. These initiatives may help build regional training capacity and reduce reliance on visiting experts. However, the existing evidence base is currently limited, as most reports were descriptive and short-term, which limits the ability to judge the durability and broader applicability of horizontal models.

### Expanding Access Through Virtual and Simulation-Based Education

Virtual and simulation-based training appeared frequently in our review, particularly after the rapid expansion of remote education during the COVID-19 pandemic. Learners in LMICs often reported improvements in their procedural skills, knowledge, and confidence after participating in tele-simulation or virtual instructional sessions [20–23]. These learning platforms can extend access to specialty training and provide practice opportunities that might not be available locally. At the same time, nearly all studies highlighted logistic barriers, such as unreliable internet access, limited protected training time, equipment failures, and uncertainty about how well simulation translates to clinical performance [20–22]. Advanced virtual reality programs also carry high costs, raising questions about sustainability [22]. These findings underscore the importance of designing virtual programs that align with available infrastructure and can be maintained locally while maintaining fiscal viability.

### Hands-On Skill Development Through In-Person and Hybrid Training

In-person targeted education and training programs continued to play an important role in hands-on procedural training. Longstanding HIC partnerships with centers in Kenya, Eritrea, and Vietnam reported increased surgical autonomy and academic engagement among local surgeon participants [25, 26, 30]. Similarly, structured models such as the IGOT’s Surgical Management and Reconstructive Training (SMART) courses have provided repeated, hands-on surgical education in low-resource contexts. The IGOT SMART courses have been held in multiple locations, including Tanzania, Nigeria, Nepal, Mexico, and Cuba [6, 31]. Importantly, SMART is explicitly designed to be locally adaptable: curricula are developed in response to host-institution identified needs and incorporate case-based discussions that center management decisions around the resources available in participants’ home clinical settings [7]. The course curriculum is also tailored to limited technology and locally-available implants, emphasizing limb-salvage soft-tissue techniques that can be performed without loupes, an operating microscope, microvascular instruments, or other high-resource equipment that may be unavailable at many LMIC training sites [7]. In-country iterations further reflect local tailoring. For example, the Tanzania course was modeled after the San Francisco program but expanded to include additional modules relevant to regional practice, such as fracture management principles and deformity correction using Ilizarov techniques, and the Nepal course incorporated local faculty with an explicit ‘train-the-trainers’ focus, facilitating a transition of leadership toward host surgeons over time [7]. Together, this combination of repeated, long-term engagement and in-country delivery is consistent with prior work showing that sustained engagement is essential for meaningful skills transfer. However, these programs also highlighted the challenges of in-person exchanges. Limited equipment, inconsistent infrastructure, and the logistical demands of international travel can restrict the number of learners who participate and affect the reliability of training over time [26, 30].

Hybrid programs that paired virtual preparation followed by short in-person workshops reported encouraging early results. Participants often reported improved knowledge and described changes in clinical management after these programs [27, 28]. Hybrid formats may allow broader participation while preserving hands-on practice, but the studies to date are small and rarely examine long-term outcomes. As a result, their scalability and sustainability remain uncertain.

### Equity, Local Autonomy, and Reciprocity in Educational Partnerships

Concerns about equity, autonomy, and the distribution of benefits were noted across several studies. Peruvian surgeons, for example, described feeling pressured to adopt external standards that did not fully reflect local realities [14], and neurosurgeons in Southeast Asia reported decreased access to surgical cases and research opportunities when hosting visiting trainees [15]. These observations are consistent with broader critiques of global surgery partnerships, particularly regarding authorship, control over educational agendas, and the degree to which collaborations genuinely support local needs. None of the included studies directly assessed the perspectives of LMIC learners on potential bidirectional exchanges into higher-resource settings, despite the emphasis on reciprocity in recent partnership frameworks.

### Funding Transparency and Sustainability of Educational Partnerships

The limited and inconsistent reporting of funding mechanisms across studies highlights an important gap in the global surgical education literature. Transparent reporting of program financing is essential for evaluating the feasibility and long-term sustainability of educational initiatives, particularly in low-resource settings where external funding and institutional support often determine whether programs can be maintained. Because only a small proportion of studies described identifiable funding sources, the financial structures supporting many educational partnerships remain unclear. Incomplete reporting of program financing limits the ability to assess sustainability across educational models. Improved reporting of funding structures and resource requirements would enable future collaborating institutions to better identify potential funding pathways and design more sustainable global orthopaedic education programs.

### Limitations

This review has several limitations. The studies we identified varied widely in design, sample size, and reporting detail, which makes it difficult to compare educational models directly or draw firm conclusions about their relative impact. Many studies focused on short-term outcomes, such as changes in perceived confidence or knowledge regarding specific procedures, rather than objective measures of skill retention or impact on patient care. Learner perspectives were also inconsistently reported and were often limited to small cohorts or single-institution partnerships, which may not reflect the experiences of trainees in other regions. Additionally, in our review we did not find any studies reporting partnerships amongst two or more LMICs. The opportunity to explore the relevance of such partnerships amongst surgical trainers and trainees with similar resource availability and spectrum of clinical pathology remains largely undocumented. Although our intent was to assess models relevant to orthopaedic education, several included studies involved mixed surgical specialties, which may reduce the specificity of some findings for orthopaedics [13–15, 21, 27]. Finally, despite using multiple databases, it is possible that relevant studies published in languages other than English or indexed elsewhere were not captured. These limitations should be considered when interpreting the results of this review.

### Implications for Future Research

Future research would benefit from prospective, multi-institutional evaluations of global surgical education partnerships that incorporate standardized outcome measures extending beyond learner perceptions to include objective technical skill acquisition, longitudinal skill retention, and patient-level clinical outcomes. Increased representation of studies led by or co-designed with LMIC investigators will be important to better reflect locally defined priorities and contextual barriers. Comparative evaluations of horizontal (LMIC-LMIC), vertical (HIC-LMIC), and hybrid collaboration models may help clarify which program structures provide the greatest educational and capacity-building benefit across different resource settings. Longer follow-up periods, improved reporting of funding structures and program costs, and formal assessment of reciprocity, authorship equity, and leadership distribution would further strengthen understanding of partnership sustainability and ethical implementation.

## Conclusion

Despite the variability in study designs, several consistent themes emerged regarding how learners in low-resource settings view international educational exchanges. Trainees and practicing surgeons alike valued opportunities that offered structured teaching, subspecialty exposure, and access to higher-volume environments. Virtual, hybrid, horizontal, and in-person models each demonstrated strengths but also faced predictable challenges related to local infrastructure, high cost, and inequities among LMIC and HIC partnerships. Concerns about agency and reciprocity were evident in multiple studies, underscoring the need for partnerships that are designed with local priorities at the forefront. Moving forward, global orthopaedic educational and training programs in HICs will likely benefit from developing closer collaboration with LMIC institutions, more rigorous evaluation of the outcomes, impact and sustainability of educational and training programs, and developing long-term strategies that support building local capacity and leadership in LMICs. Greater transparency in reporting funding mechanisms and resource requirements will also be important to facilitate replication and long-term sustainability of successful educational partnerships. Furthermore, the potential impact of educational and training models between surgeons from limited-resourced surgeons practicing in different parts of the globe, i.e. South-South partnerships remain largely unknown. Thoughtfully designed exchange programs have the potential to strengthen surgical education and improve access to musculoskeletal care not only in underserved settings but also positively impact the professional development of the trainees and surgeons irrespective of resource availability.

## Key References

- Lu PM, Mansour R, Qiu MK, Biraro IA, Rabin TL. Low- and Middle-Income Country Host Perceptions of Short-Term Experiences in Global Health: A Systematic Review. Acad Med. 2021;96(3):460–469. 
  - This systematic review synthesizes host perspectives on short-term global health and educational exchanges, offering an important lens on equity, ethics, and power dynamics. Its findings provide essential context for interpreting learner-reported preferences and concerns described in this review
- Conway DJ, Coughlin R, Caldwell A, Shearer D. The Institute for Global Orthopedics and Traumatology: A Model for Academic Collaboration in Orthopedic Surgery. Front Public Health. 2017;5:146. 
  - This paper describes the development and structure of the IGOT as a model for sustained academic collaboration and in-country orthopaedic education, including educational initiatives such as the SMART courses.
- Shapiro LM, Welch JM, Chatterjee M, et al. A Framework and Blueprint for Building Capacity in Global Orthopaedic Surgical Outreach. J Bone Jt Surg. 2023;105(3):e10.
  - This paper proposes an orthopaedic-specific framework for designing ethical and sustainable outreach programs and informs interpretation of themes related to local leadership, reciprocity, and long-term engagement across the included studies.
- Asfaw ZK, Todd R, Abasi U, et al. Use of virtual platform for delivery of simulation-based laparoscopic training curriculum in LMICs. Surg Endosc. 2023;37(2):1528–1536.
  - This quasi-experimental study evaluates virtual, simulation-based laparoscopic training curriculum delivered in LMIC settings, demonstrating short-term improvements in technical performance and supporting the feasibility of tele simulation-based surgical education.

## Appendix A

Full Search Strategy

**PubMed**

Date of Search: September 2025.

Number of results: 70.

Query:

("Education, Medical"[Mesh] OR "medical education"[tiab] OR "Clinical Competence"[Mesh] OR "clinical competence"[tiab] OR "clinical skills"[tiab]) AND ("Education, Distance"[Mesh] OR "distance education"[tiab] OR "remote learning"[tiab] OR "distance learn*"[tiab] OR "observership"[tiab] OR "in-person"[tiab] OR "in person"[tiab] OR "educational exchange"[tiab] OR "exchange programs"[tiab] OR webinar*[tiab] OR Youtube[tiab] OR "remote learning"[tiab]) AND ("Developing Countries"[Mesh] OR "Low and Middle Income Countries"[tiab] OR "LMICs"[tiab] OR "developing countries"[tiab] OR "low-resource"[tiab] OR "high-resource"[tiab]) AND (perception*[tiab] OR preference*[tiab] OR experience*[tiab] OR opinion*[tiab] OR perspective*[tiab]).

**Ovid Medline**

Date of Search: September 2025.

Number of results: 95.

Query:

(Exp education, medical/OR exp clinical competence/OR (medical education OR (clinical adj2 (competenc* OR skill*)))).ti,ab,kf. AND (Exp education, distance/OR ((distance OR remote) adj2 (learn* OR education) OR observership* OR in?person OR educational exchange OR exchange program* OR webinar* OR YouTube)).ti,ab,kf. AND (Exp developing countries/OR (“Low and middle income countr*” OR LMICs OR developing countr* OR low?resource* OR high?resource*)).ti,ab,kf. AND (Perception* or preference* or experience* or opinion* or perspective*).ti,ab,kf.

**Scopus**

Date of Search: September 2025.

Number of results: 101.

Query:

TITLE-ABS-KEY((medical education OR (clinical W/2 competenc* OR skill*)) AND ((distance OR remote) W/2 (learn* OR education) OR observership* OR in?person OR “educational exchange” OR “exchange program*” OR webinar* OR YouTube) AND (“Low and middle income countr*” OR LMICs OR “developing countr*” OR “low?resource*” OR “high?resource*”) AND (Perception* OR preference* OR experience* OR opinion*)).

**CINAHL (EBSCOhost)**

Date of Search: September 2025.

Number of results: 58.

Query:

(MH "Education, Medical + " OR MH "Clinical Competence + " OR XB (medical education OR (clinical N2 (competenc* OR skill*)))) AND (XB ((distance OR remote) N2 (learn* OR education) OR observership* OR in?person OR educational exchange OR exchange program* OR webinar* OR YouTube)) AND (MH "Developing Countries" OR XB (“Low and middle income countr*”* OR LMICs OR developing countr* OR low?resource* OR high?resource*)) AND (XB (Perception* or preference* or experience* or opinion* or perspective*)).

**ERIC (EBSCOhost)**

Date of Search: September 2025.

Number of results: 44.

Query:

(DE "Medical Education" OR TI (medical education OR (clinical N2 (competenc* OR skill*) OR AB (medical education OR (clinical N2 (competenc* OR skill*)))) AND (TI ((distance OR remote) N2 (learn* OR education) OR observership* OR in?person OR educational exchange OR exchange program* OR webinar* OR YouTube) OR AB ((distance OR remote) N2 (learn* OR education) OR observership* OR in?person OR educational exchange OR exchange program* OR webinar* OR YouTube)) AND (DE "Developing Countries" OR TI (“Low and middle income countr*” OR LMICs OR developing countr* OR low?resource* OR high?resource*) OR AB (“Low and middle income countr*” OR LMICs OR developing countr* OR low?resource* OR high?resource*)) AND (TI (Perception* or preference* or experience* or opinion* or perspective*) OR AB (Perception* or preference* or experience* or opinion* or perspective*)).

**Cochrane Central Register of Controleld Trials (CENTRAL)**

Date of Search: April 9, 2025.

**Table:**

ID	Search Strategy	Results
#1	MeSH descriptor: [Education, Medical] explode all trees	4861
#2	MeSH descriptor: [Clinical Competence] explode all trees	5441
#3	(medical education OR (clinical NEAR/2 (competenc* OR skill*))):ti,ab,kw	40,248
#4	#1 OR #2 OR #3	40,743
#5	MeSH descriptor: [Education, Distance] explode all trees	372
#6	(((distance OR remote) NEAR/2 (learn* OR education) OR observership* OR in?person OR "educational exchange" OR (exchange NEXT program*) OR webinar* OR YouTube)):ti,ab,kw	8877
#7	#5 OR #6	8877
#8	MeSH descriptor: [Developing Countries] explode all trees	1271
#9	(((Low and middle income NEXT countr*) OR LMICs OR (developing NEXT countr*) OR low?resource* OR high?resource*)):ti,ab,kw	10,411
#10	#8 OR #9 10,411	10,411
#11	((Perception* or preference* or experience* or opinion* or perspective*)):ti,ab,kw	228,865
#12	#4 AND #7 AND #10 AND #11	6

## References

[1] Roberts HJ, Albright PD, Shearer DW, et al. Motivations and impact of international rotations in low- and middle-income countries for orthopaedic surgery residents: are we on the same page? Am J Surg. 2021;221(2):245–53. 10.1016/j.amjsurg.2020.08.046. 33092782 10.1016/j.amjsurg.2020.08.046

[2] Roberts HJ, Coss N, Urva M, et al. Host perspectives of high-income country orthopaedic resident rotations in low and middle-income countries. J Bone Joint Surg Am. 2022;104(18):1667–74. 10.2106/JBJS.22.00050. 35778996 10.2106/JBJS.22.00050

[3] World Bank. World Bank Country and Lending Groups [Internet]. The World Bank. 2024. Available from: https://datahelpdesk.worldbank.org/knowledgebase/articles/906519-world-bank-country-and-lending-groups

[4] Wassef DW, Holler JT, Pinner A, et al. Perceptions of orthopaedic volunteers and their local hosts in low- and middle-income countries: are we on the same page? J Orthop Trauma. 2018;32(7):S29-34. 10.1097/BOT.0000000000001297. 30247397 10.1097/BOT.0000000000001297

[5] Lu PM, Mansour R, Qiu MK, Biraro IA, Rabin TL. Low- and middle-income country host perceptions of short-term experiences in global health: a systematic review. Acad Med. 2021;96(3):460–9. 10.1097/ACM.0000000000003867. 33298696 10.1097/ACM.0000000000003867

[6] Turissini M, Mercer T, Baenziger J, et al. Developing ethical and sustainable global health educational exchanges for clinical trainees: implementation and lessons learned from the 30-year Academic Model Providing Access to Healthcare (AMPATH) partnership. Ann Glob Health. 2020;86(1):137. 10.5334/aogh.2782. 33178558 10.5334/aogh.2782 PMC7597575

[7] Conway DJ, Coughlin R, Caldwell A, Shearer D. The institute for Global Orthopedics and Traumatology: a model for academic collaboration in orthopedic surgery. Front Public Health. 2017;5:146. 10.3389/fpubh.2017.00146. 28713803 10.3389/fpubh.2017.00146 PMC5491941

[8] Shapiro LM, Welch JM, Chatterjee M, et al. A framework and blueprint for building capacity in global orthopaedic surgical outreach. J Bone Joint Surg. 2023;105(3):e10. 10.2106/JBJS.22.00353. 35984012 10.2106/JBJS.22.00353 PMC10760412

[9] Brown K, Flores MJ, Haonga B, et al. Best practices for developing international academic partnerships in orthopaedics. J Bone Joint Surg Am. 2024;106(10):924–30. 10.2106/JBJS.23.00626. 37851955 10.2106/JBJS.23.00626 PMC11593968

[10] Sors TG, O’Brien RC, Scanlon ML, et al. Reciprocal innovation: a new approach to equitable and mutually beneficial global health partnerships. Glob Public Health. 2023;18(1):2102202. 10.1080/17441692.2022.2102202. 35877989 10.1080/17441692.2022.2102202 PMC9873831

[11] Tricco AC, Lillie E, Zarin W, et al. PRISMA extension for scoping reviews (PRISMA-ScR): checklist and explanation. Ann Intern Med. 2018;169(7):467–73. 10.7326/M18-0850. 30178033 10.7326/M18-0850

[12] Covidence systematic review software,. Available at www.covidence.org.

[13] Khan HJ, Ghumman AK, Yunus T. Bridging the gap: addressing barriers in bariatric surgery training in Pakistan. Cureus. 2025. 10.7759/cureus.87613. 40786422 10.7759/cureus.87613 PMC12334076

[14] LaGrone LN, Isquith-Dicker LN, Huaman Egoavil E, et al. Surgeons’ and trauma care physicians’ perception of the impact of the globalization of medical education on quality of care in Lima, Peru. JAMA Surg. 2017;152(3):251. 10.1001/jamasurg.2016.4073. 27893012 10.1001/jamasurg.2016.4073

[15] Lepard JR, Corley J, Sankey EW, et al. Training neurosurgeons in Myanmar and surrounding countries: the resident perspective. World Neurosurg. 2020;139:75–82. 10.1016/j.wneu.2020.03.114. 32251819 10.1016/j.wneu.2020.03.114

[16] Agrawal R, Mishra SK, Mishra A, et al. Role of telemedicine technology in endocrine surgery knowledge sharing. Telemed J E Health. 2014;20(9):868–74. 10.1089/tmj.2013.0164. 25078673 10.1089/tmj.2013.0164

[17] Martins RS, Ukrani RD, Raza Raja MH, et al. Peer-taught virtual research workshops for surgical residents: Protocol for a novel and sustainable solution to improving surgical research in Pakistan. JPMA J Pak Med Assoc. 2021;71(1):S130–5. 33582740

[18] Quraishi N, Quraishi S. Otolaryngology education and training in the COVID-19 and post-COVID-19 digital era: a developing world perspective. Curr Opin Otolaryngol Head Neck Surg. 2021;29(3):225–9. 10.1097/MOO.0000000000000709. 33896912 10.1097/MOO.0000000000000709

[19] Fagan JJ. Editorial: otolaryngology education and training in the COVID-19 and post-COVID-19 digital era: a developing world perspective. Curr Opin Otolaryngol Head Neck Surg. 2021;29(3):219–20. 10.1097/MOO.0000000000000715. 33896911 10.1097/MOO.0000000000000715

[20] Theodorakopoulou E, Goutos I, Mason K, Ghanem AM, Myers S. <article-title update="added">London calling Gaza: the role of international collaborations in the globalisation of postgraduate burn care education. Scars, Burns & Healing. 2019;5:2059513119830519. 10.1177/2059513119830519. 10.1177/2059513119830519 PMC6381431 30815281

[21] Asfaw ZK, Todd R, Abasi U, et al. Use of virtual platform for delivery of simulation-based laparoscopic training curriculum in LMICs. Surg Endosc. 2023;37(2):1528–36. 10.1007/s00464-022-09438-w. 35852623 10.1007/s00464-022-09438-w

[22] Woodard S, Kleiman K. Virtual reality simulation–based training in image-guided breast intervention in low- and middle-income countries. AJR Am J Roentgenol. 2024;223(2):e2431236. 10.2214/AJR.24.31236. 38775435 10.2214/AJR.24.31236

[23] Taekman JM, Foureman MF, Bulamba F, et al. A novel multiplayer screen-based simulation experience for African learners improved confidence in management of postpartum hemorrhage. Front Public Health. 2017;5:248. 10.3389/fpubh.2017.00248. 29018791 10.3389/fpubh.2017.00248 PMC5623004

[24] Schönfeld CL, Nyaga P, Onyango OM, Klauss V, Kampik A, Kollmann M. Benefits, limitations and outcomes of vitreoretinal surgery, Nairobi, Kenya: 8-year experience. Acta Ophthalmol. 2012;90(3):e244-245. 10.1111/j.1755-3768.2011.02153.x. 21649875 10.1111/j.1755-3768.2011.02153.x

[25] Jungehülsing M, Markmiller U, Schröder U, Book A, Teclu A, Stennert E. MEDCARE for People in Eritrea: Ein Bericht über 9 Jahre Hilfe zur Selbsthilfe. HNO. 2019;67(7):502–9. 10.1007/s00106-019-0690-y. 31165200 10.1007/s00106-019-0690-y

[26] Merrell JC, Tien NV, Son NT, et al. <article-title update="added">Introduction of microsurgery in Vietnam by a charitable organization: a 15-year experience: Plast Reconstr Surg. 2007;119(4):1267–73. 10.1097/01.prs.0000254544.65054.95. 17496600 10.1097/01.prs.0000254544.65054.95

[27] Moreira DC, Gajjar A, Patay Z, et al. Creation of a successful multidisciplinary course in pediatric neuro‐oncology with a systematic approach to curriculum development. Cancer. 2021;127(7):1126–33. 10.1002/cncr.33350. 33259071 10.1002/cncr.33350

[28] Siddharthan T, Jackson P, Argento AC, et al. A pilot program assessing bronchoscopy training and program initiation in a low-income country. J Bronchol Interv Pulmonol. 2021;28(2):138–42. 10.1097/LBR.0000000000000721. 10.1097/LBR.0000000000000721 33105417

[29] Costa F, Servadei F. Webinar during COVID-19 pandemic: necessity or uncontrolled phenomena? World Neurosurg. 2021;154:186. 10.1016/j.wneu.2021.07.087. 34583482 10.1016/j.wneu.2021.07.087 PMC8529613

[30] Schoenfeld AJ, Herzog JP, Dunn JC, Bader JO, Belmont PJ. Patient-based and surgical characteristics associated with the acute development of deep venous thrombosis and pulmonary embolism after spine surgery. Spine. 2013;38(21):1892–8. 10.1097/BRS.0b013e31829fc3a0. 23778367 10.1097/BRS.0b013e31829fc3a0

[31] Institute for Global Orthopaedics and Traumatology (IGOT). IGOT Tanzania SMART Course. UCSF Department of Orthopaedic Surgery; 2023 Jul 12. Available from: https://orthosurgery.ucsf.edu/about/news-media/institute-global-orthopaedics-and-traumatology-igot-tanzania-smart-course

